# From curfews to skipping 8 am classes: 100 years of university student sleep culture

**DOI:** 10.1093/sleep/zsaf183

**Published:** 2025-07-13

**Authors:** Michael K Scullin, Jason R Carter

**Affiliations:** Department of Psychology and Neuroscience, Baylor University, Waco, TX, United States; Department of Health, Human Performance, and Recreation, Baylor University, Waco, TX, United States

**Figure 1.**
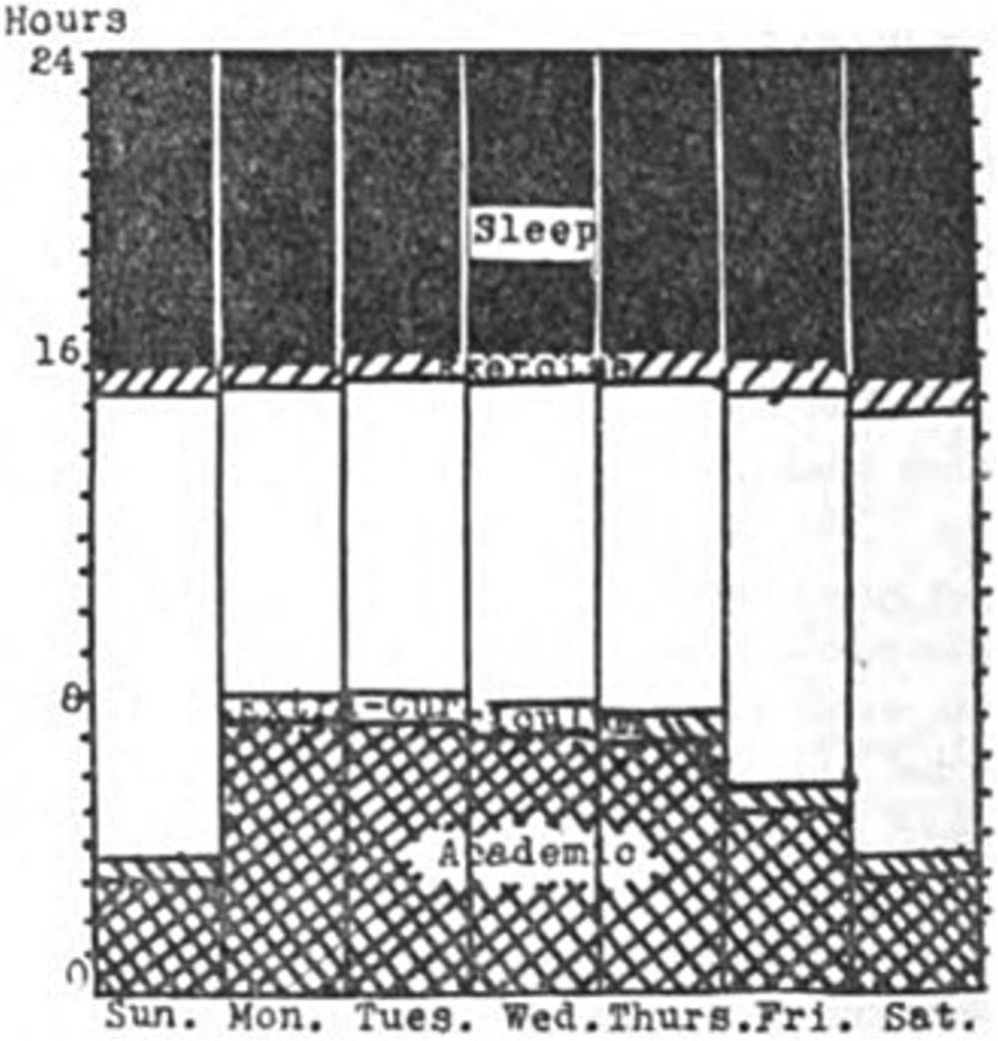
Time-use diary data for sleep, exercise, extracurricular, and academic activities from 503 students at Vassar College during the spring semester of 1925 [1]. The unaccounted time (white bars) includes eating, dressing, chapel, and an estimated 6 hours of leisure activity. The figure is reprinted from a public domain source, courtesy of HathiTrust.

In 1925, half of Vassar College students maintained a time-use diary for a semester, including sleep duration estimates [1]. At the time, Vassar College was a highly selective, female-only college with most students living on campus. Digital media did not exist, and students had evening curfews. In this context, one century ago, Vasser College students reported sleeping 7.9 hours/night, with minimal fluctuations between weekday and weekend nights (Figure 1). At nearby Mount Holyoke College, students averaged 8.3 hours/night [1]. How sleep patterns have changed amongst university students over the last 100 years.

Today’s estimates are that 60% of university students have poor sleep quality [2], half of the students sleep fewer than 7 hours/night [3], and 27% meet criteria for clinical insomnia [4]. Similar statistics have emerged across a range of higher education settings, but the reliance on self-report measures and cross-sectional designs has limited our understanding of how and why sleep patterns evolve during college. With few exceptions [5–7], when objective sleep tracking has been used in college students, the sample sizes have been small or the monitoring period has been brief.

In this issue of SLEEP, Soon et al. [8] used Oura Ring 3 devices to monitor 638 freshmen students during their first 20 weeks at the National University of Singapore (NUS). Their study details several surprising—and sometimes alarming—results. For example, the *average* bedtime for students living on campus was 02:04 am, approximately 2 hours later than estimates in studies that relied on students’ self-reported bedtimes [9–11]. On weekday nights, NUS students went to bed only 12–17 minutes earlier than on weekend nights, suggesting milder intra-individual variability and fewer sleep recovery attempts than studies that documented severe social jetlag in college students [11–13].

Why are NUS students going to bed so late? Existing frameworks would suggest late bedtimes are due to biological dispositions (evening chronotypes), academic stressors, and social obligations [14]. Though the study did not include genotyping or traditional measures of circadian timing (e.g. dim light melatonin onset), the data did include self-reported chronotypes and estimates of study time. Only 36.5% of students were classified as “evening” chronotypes on the Morningness-Eveningness Questionnaire, and students reported spending only 27.5%–30.4% of their time on studying in the 4 hours before bedtime. These data challenged the common claim, often from students, that academic work and/or the innate desire to stay up late prevent one from adopting earlier bedtimes.

The late bedtimes in NUS students appeared driven by socio-cultural influences. In contrast to Vassar College’s culture 100 years ago (e.g., evening curfew, no digital media) [1], time usage data in current NUS students indicated that social and digital leisure activities dominated in the 4 hours before bedtime. Living on campus further exacerbated bedtimes by 37 minutes/night, particularly for students who engaged in more late-night social activities, which is sometimes required for maintaining hostel (dormitory) residency. Off-campus students who engaged in more digital leisure activities also showed more delayed bedtimes [15].

While the bedtime findings could have been captured with a cross-sectional design, longitudinal monitoring allowed Soon et al. [8] to identify changes in sleep duration across the semester. Total sleep time was greatest during vacation weeks and reading weeks, which is understandable because these weeks did not involve scheduled classes and may be less stressful. However, sleep duration also changed significantly across instructional weeks, with a marked and steady reduction of weekday sleep duration to approximately 6.2 hours/night during the first four weeks of the semester when homesickness, adverse experiences, and other transition-related stressors have been shown to negatively impact mental health and well-being [16, 17]. Many educators, including the authors of this editorial, would predict that students’ sleep would continue to shorten or worsen in quality across the semester as academic stressors accumulate [18, 19]. However, NUS students showed the reverse: they progressively *increased* their sleep duration after their mid-term examinations. By the final instructional week of the semester, students were sleeping nearly as much as during the reading weeks.

How can freshmen students increase their sleep during a semester? Conventional wisdom is that early class start times prevent students from sleeping later in the morning [7, 20, 21]; therefore, students would either need to improve their sleep efficiency, advance their bedtimes, or increase their daytime napping. None of these sleep behaviors changed dramatically during the semester in Soon et al.’s study [8]. Instead, NUS students delayed their weekday morning wake times by nearly an hour from Week 1 to Week 14 (i.e. the last instructional week), despite having early class times. On 50% of days with 8 am classes, students slept past their class start time. The inflection point was the mid-term examination period. The median wake time was 07:31 am before mid-terms and 07:59 am after mid-terms. By the end of the semester, students were waking up on instructional weekdays as late as they did during reading weeks, when no classes were scheduled. While Soon et al.’s [8] study did not collect data on why students’ sleep timing changed; one plausible explanation is that students prioritized sleep over in-person class attendance. Students might choose sleep over attending class if attendance were not compulsory, if the class lecturing was not helpful to learning, and/or if the classes were recorded and could be watched later in the day at accelerated speeds.

Soon et al.’s [8] study is the latest work from a center that has been a leader in research on sleep and learning. Their work has demonstrated conditions under which napping is better for learning than cramming [22], that sleep education by itself is insufficient for changing sleep behaviors [23], and that early morning classes can lead to shorter sleep durations and worse academic performance [20], amongst many other contributions. These are welcome advances to a field that has historically been characterized either by cross-sectional studies with self-report outcomes or laboratory studies with artificial learning materials and studying conditions.

Soon et al.’s [8] findings arise from a single, highly selective university in Singapore, and therefore may not generalize to universities of different student compositions, sizes, or countries/cultures, as implied by the sleep data at Vassar College 100 years ago [1]. As with most longitudinal studies, adherence to wearing the Oura rings and reporting on time usage dropped from approximately 90% in Week 1 to approximately 60%–70% by Week 14. These rates were reasonable considering the study population and burden of repeated assessments, but imperfect for informing generalizability. Nevertheless, the study’s large sample size, longitudinal design, and objective sleep tracking represent a clear methodological advance on the existing literature and highlight how cultural, social/digital, and systemic factors can influence sleep in university students. Given students’ proclivity to sleep past 8 am classes, it is time to recognize that class start times are not only an issue for high schools [19]. University administrators concerned with student success, mental health, and retention might reconsider the timing of undergraduate classes and assessment deadlines, particularly for first-year students.
